# Adolescent parenthood and HIV-infection in South Africa—Associations with child cognitive development

**DOI:** 10.1371/journal.pgph.0000238

**Published:** 2022-05-02

**Authors:** Kathryn J. Steventon Roberts, Lorraine Sherr, Katharina Haag, Colette Smith, Janina Jochim, Elona Toska, Marguerite Marlow, Lucie Cluver

**Affiliations:** 1 Institute for Global Health, University College London, London, United Kingdom; 2 Department of Social Policy and Intervention, University of Oxford, Oxford, United Kingdom; 3 Centre for Social Science Research, University of Cape Town, Cape Town, South Africa; 4 Department of Sociology, University of Cape Town, Cape Town, South Africa; 5 Stellenbosch University, Stellenbosch, South Africa; 6 Department of Psychiatry and Mental Health, University of Cape Town, Cape Town, South Africa; Barcelona Institute for Global Health: Instituto de Salud Global Barcelona, SPAIN

## Abstract

HIV, both directly and indirectly, impacts child development outcomes. The most severe impacts are for children infected with HIV, and those exposed but uninfected are also shown to have challenges–though less severe. However, little is known regarding the development of children born to adolescent mothers affected by HIV. This study aims to examine cognitive development for children born to adolescent mothers, comparing those children living with HIV, those HIV exposed and uninfected (HEU) and those HIV unexposed (HU). Analyses utilise cross-sectional data from 920 adolescent mother (10–19 years)-first born child dyads residing in the Eastern Cape Province, South Africa. Participants completed detailed study questionnaires inclusive of validated and study specific measures relating to sociodemographic characteristics, HIV, and maternal and child health. Trained assessors administered standardised child development assessments (using the Mullen Scales of Early Learning) with all children. Chi-square tests and ANOVA tests were used to explore maternal and child characteristics according to child HIV status (HIV, HEU, HU) on cognitive development. Linear regression models were used to explore the cross-sectional associations between child HIV status and child cognitive development. 1.2% of children were living with HIV, 20.5% were classified as being HEU and, 78.3% were classified as HU. Overall, children living with HIV were found to perform lower across developmental domains compared to both HEU and HU groups (composite score of early learning: 73.0 vs 91.2 vs. 94.1, respectively: *F* = 6.45, p = 0.001). HEU children on average scored lower on all developmental domains compared to HU children, reaching significance on the gross motor domain (p<0.05). Exploratory analyses identified maternal education interruption as a potential risk factor for lower child cognitive development scores and, higher maternal age to be protective of child cognitive development scores. These exploratory findings address a critical evidence gap regarding the cognitive development of children born to adolescent mothers affected by HIV in South Africa. Analyses identify stepwise differences in the average scoring on child cognitive development domains according to child HIV status among children born to adolescent mothers affected by HIV; with children living with HIV performing worse overall. Young mothers and their children may benefit from adapted interventions aimed at bolstering child development outcomes. Targeted programming particularly among younger adolescent mothers and those experiencing education interruption may identify those families, particularly in need. Attention to maternal continuity of education and age of conception may be interventions to consider.

## Introduction

While there has been a growth of attention given to adolescents within the global HIV response in recent years, adolescent mothers and their children remain mostly disregarded within existing literature, funding, and programmatic responses [1]. Over the coming decade, sub-Saharan Africa will become home to over 16 million adolescent mothers (10–19 years) and thus to more children born to adolescent mothers [2]. The wellbeing and developmental trajectory of adolescent mothers and their 3 children is critical not only to individuals and the efforts towards the elimination of HIV [1, 3, 4], but also to the success of the sub-Saharan African region as a whole [1, 2, 5].

The prevalence rate of adolescent pregnancy within South Africa is one of the highest in the world—almost a fifth of female adolescents’ report experiencing pregnancy [6]. Adolescent motherhood has previously been found to be associated with adverse pregnancy outcomes [7], child developmental delay and, poor health outcomes among children [8–11]. Children not reaching their developmental potential within early childhood may have adverse outcomes across the life course and could in turn lead to cyclical disadvantage, which has implications for the prosperity and future success of individuals, their families and, the communities in which they reside [12, 13]. South Africa has the largest HIV epidemic globally [14] and, female adolescents (15–19 years) remain disproportionately affected [15, 16], with incidence rates of HIV almost five times greater compared to male adolescents [14]. Given the high incidence rates of both adolescent motherhood and HIV within South Africa, it is critical that we develop an understanding of the development of children born with the combined experience of being born to adolescent mothers affected by HIV. An understanding of the realities of this key population will inform how best to support this group and ensure adequate policy and programming that is reflective of need [1].

The literature on adolescent mothers and the literature on maternal HIV infection have both demonstrated child development impacts. However, there is a dearth of understanding on the experiences when both co-occur. For children born to adolescent mothers living with HIV, implications for child cognitive development may be compounded. There is a strong body of literature highlighting the adverse impacts (both direct and indirect) of HIV on children inclusive of developmental delay [17, 18]. Given the rise in programming focusing on vertical transmission prevention in recent years, fewer children are now living with HIV and there is a growing group of children who are HIV exposed but uninfected [19]. Previous investigations within both high income and, low-middle income countries, have identified worse development outcomes of children who are HIV infected and HIV exposed uninfected (HEU) compared to children who are HIV unexposed (HU) [20].

Early child development is a period of rapid and complex growth and skill development that is impacted by a multitude of factors inclusive of biological, social, and environmental factors [21]. Among children living with HIV and those children who are HEU, cognitive deficits may be due to the direct physical and structural effects of HIV on the nervous system and cognition [22], exposure to treatment and, broader factors related to child and/or maternal HIV infection [20]. Indirectly, HIV infection and HIV exposure (i.e. maternal HIV infection) may impact the environment that the child is exposed to, as familial HIV infection has been found to be associated with a greater likelihood of poverty, food insecurity, caregiver illness, mental health problems, distracted parenting, lone parenting, bereavement [23]. In addition to HIV playing a role within the developmental outcomes of children, the neurological development of children has also been found to be impacted by quality of caregiving inclusive of knowledge of child rearing, early years stimulation and, parental mental health (which may be poorer in adolescents when compared to adult mothers [24] and, has been found to be worse among adolescent mothers living with HIV compared to adolescent mothers not living with HIV) [25]. Likewise, adverse child development outcomes have been found to be mitigated by interventions promoting early years stimulation and increased quality of caregiving [26]. Yet, the neurological profile of children born to adolescent mothers affected by HIV (both living with HIV and, living within HIV affected communities) remains underexplored and we are yet to understanding the implications of HIV exposure for these children.

The current COVID-19 pandemic has highlighted particular vulnerabilities within our communities. Fiscal capacity and general access to resources may be reduced and, communities have been challenged by increased food insecurity, reduced economic opportunities, rising levels of violence and disruptions to healthcare (i.e., contraceptive services, access to HIV care). The latter leading to an increase in the number of unintended pregnancies among adolescents [2, 27, 28]. As such, understanding the needs of this potentially vulnerable population and their children has become increasingly important to understanding how adolescent mothers and their children, especially given a background of familial HIV infection, may be best supported by policy and programming.

These exploratory analyses investigate the relationship between the HIV status of children born to adolescent mothers (HIV, HEU and HU) and child cognitive development outcomes. Based on previous literature, we hypothesised that the cognitive performance of children born to adolescent mothers would be worst in children living with HIV, followed by children classified as HEU and, then HU children.

## Methods

### Participants and procedure

Data utilised within these analyses were drawn from a large cohort study of adolescent and young mothers and their children residing in the Eastern Cape province of South Africa (n = 1046; *Helping Empower Youth Brought up in Adversity with their Babies and Young Children [HEY BABY]* study). Young mothers with at least one living child were interviewed between March 2018 and July 2019. Participants were recruited from both rural and peri-urban health districts utilising six parallel sampling strategies developed with local experts and an advisory group of adolescent mothers. Recruitment channels included: all known health facilities within the district (n = 73), secondary schools (randomly selected; n = 43), maternity obstetric units (n = 9), referrals by service providers and social workers, neighbouring adolescents of participants (to reduce stigma) and, referrals by adolescent mothers themselves. Piloting of data collection tools was undertaken with adolescent mothers (n = 9) and adolescents living with HIV (n = 25).

Only data for adolescent mothers (who had given birth between 10–19 years of age), and their first-born children (0–68 months) were included within subsequent analyses (n = 920 adolescent mother-child dyads). Participants were excluded from analyses if they were older than 19 years at the birth of their first child (n = 40), children were above 68 months of age (in line with the valid age range for the child development assessments used within this study (n = 30), or the HIV status on the child was unknown or unclear (n = 52). Second and third born children were also excluded from these analyses.

### Ethics statement

Informed voluntary consent was obtained from all participants and, in the instance when an adolescent was under 18 years of age, their adult caregivers. Additional consent was obtained from the primary caregiver of the infant if adolescent mothers identified that they were not the main caregiver of their child. The majority, 85.8% (789/920) of adolescent mothers in the included sample, reported that they were the main caregiver of their child. However, other main caregivers identified included the child’s father (0.43%; 4/920), the maternal grandmother (7.9%; 73/920), partner–not the child’s father (0.43%; 4/920), someone else in the family (5.1%; 47/920) or someone in the community (0.33%; 3/920). Ethical approvals were obtained from the Universities of Oxford (R48876/RE002) and Cape Town (HREC 226/2017) as well as University College London (14795/001). Additional local approvals and permissions were obtained from the Provincial Departments (Eastern Cape, South Africa) of Health, Education, and Social Development as well as participating health and education facilities.

### Measures

Analyses utilise cross-sectional data from a range of self-report measures and a standardised child assessment. Three aspects of data were collected inclusive of; 1) detailed self-report adolescent interviews focusing on sociodemographic characteristics, physical and mental health, pregnancy, and parenting experiences, 2) caregiver report of child data and, 3) standardised child development measures (utilising the Mullen Scales of Early Learning) administered by a trained interviewer. Trained data collectors administered questionnaires utilising tablets to maintain confidentiality. Questionnaires and consent forms were completed in the participant’s language of choice (isiXhosa/English) and were translated and back-translated as appropriate.

**Sociodemographic characteristics** inclusive of age (maternal and child), age at onset of pregnancy, child biological sex and maternal relationship status were routinely collected. **Maternal HIV status** was ascertained through self-report and verified through clinical records. **Child HIV status** was ascertained through caregiver report and corroborated with child medical records (Road to Health Cards). Three groups of interest relating to child HIV status were created based on a combination of reported maternal and child HIV status (living with HIV, HIV exposed-uninfected [HEU] and, HIV unexposed [HU]). **Socioeconomic status** was assessed utilising numerous measures: **Access to necessities** was assessed though multiple items- as such, adolescent mothers were asked if they had enough food in the past week and could afford three meals daily (yes/no) and, whether they had access to a list of eight basic necessities for their both them and their children (0–8; e.g., enough clothes to keep you warm and dry). **Cash grant receipt and household employment** was assessed via maternal self-report. **Maternal education** was assessed by a single self-report item asking if mothers were at least a school grade behind where they should be according to their age (number school grades ahead/behind ranged from +2, -6), participants were classified as experiencing **maternal education interruption** if they were at least one school grade behind. **Social support** was measured using 8 items from the Medical Outcomes Study (MOS) Social Support Survey [29]. **Violence exposure** (both community and domestic) was ascertained by maternal self-report. **Maternal mode of HIV infection** (perinatal vs. postnatal) was not directly assessed in the study. This variable was derived using a logic tree based on field and clinical experience and has been used within previous explorations (see Sherr et al. 2018 [30] for full details on how the variable was derived). Adolescents who were living with HIV (n = 200) were asked to report **antiretroviral (ART) adherence** in the past week. **Any common mental disorder** was a composite measure of four measures of maternal mental health symptomology (depression [31, 32], anxiety [33, 34], trauma [35, 36], and suicidality [37, 38]). Mothers were classified as experiencing common mental disorder if they scored above the validated cut-off on any measures of mental health symptomology (depression: short form of the Child Depression Inventory [3/10] [39], anxiety: Revised Children’s Manifest Anxiety Scales [10/14] [33, 34], trauma: Child PTSD Checklist [partial trauma: >1 on each of the four PTSD domains] [35], suicidality: Mini International Psychiatric Interview for Children and Adolescents [1/5]) [37, 38].

**Child cognitive development** was assessed using the Mullen Scales of Early Learning [40]. These capture development across five domains (gross motor skills, visual reception, fine motor skills, expressive languages and, receptive language). Children completed a battery of assessments (administered by trained data collectors) for each domain. Raw scores were transformed into age standardised t-scores (range 20–80). Four of the developmental domains (visual reception, fine motor skills, expressive language and, receptive language) were combined and standardised based on validated methodology to form compositive score of generalised cognitive development (range 49–155) [40]. The gross motor skills assessment was only undertaken with children <39 months of age (n = 831/920; HIV = 9, HEU = 163, HU = 659). All children within the validated age range (0–68 months) completed assessments for the remaining four domains [40].

### Statistical analyses

Data analyses were cross-sectional and undertaken utilising STATA v.15. Summary statistics for each outcome were computed and ANVOA tests (inclusive of Tukey’s HSD post hoc testing) and chi-square tests were used to explore sample characteristics according to child HIV status (living with HIV, HIV exposed uninfected, HIV unexposed). Within exploratory analyses, linear regression models were used to investigate the associations between child HIV status and child development. Three models were run: 1) univariate analyses exploring the association between child HIV status (as well as sociodemographic characteristics) and child cognitive development scores, 2) multivariate analyses inclusive of covariates and, 3) multivariate analyses exploring possible mitigating factors within the relationship between child HIV status and, child cognitive development. Covariates were included within multivariable regression models if there were found to be associated with both or either, the predictor and outcome variables (within univariate analyses p = <0.2) or were identified as relevant within the literature [41, 42]. Sample characteristics considered as covariates (a priori) included child age, child biological sex, maternal age, relationship status, socio-economic status, maternal education, social support, violence exposure, maternal ART adherence and, maternal mental health. The number of covariates within multivariate analyses was restricted to account for the small sample of children identified as living with HIV (n = 11). Given this restriction, child age, child biological sex and number of necessities the mother could afford (as a measure of socio-economic status) were included as covariates within model 2. Maternal education interruption and maternal age were included within model 3 to explore potential mitigating factors within the relationship between child HIV status and child cognitive development scores.

## Results

### Sociodemographic characteristics

Table 1 presents sample characteristics, both maternal and child, according to child HIV status (living with HIV, being HIV exposed uninfected, being HIV unexposed). 1.2% (11/920) of children were living with HIV, 20.5% (189/920) were classified as being HEU and, 78.3% (720/920) were classified as HU. As such, the HIV transmission rate from mother to child was 5.5% (11/200). The mean age of mothers at the time of the birth of their child was 16.6 years (SD:1.5) total sample. Mothers of children who were HEU were older at the time of their birth (HEU: 17.4 years vs. HIV: 16.6 years vs. HU: 16.3 years). The mean age of mothers at baseline data collection was 18.0 years (SD:1.5). The mean age of children born to adolescent mothers at baseline was 17.9 months (SD:14.2). Children born to adolescent mothers living with HIV (living with HIV and HEU) were found to be older compared to those who were HU (HIV: 28.5 vs. HEU: 21.6 vs. HU: 16.8 months; *F* = 76.2, *p* = <0.0001). No differences were identified according to biological sex of children born to adolescent mothers (overall 48% female). Adolescent mothers of children living with HIV were less likely to be food secure (*X*^*2*^ = 8.51, *p* = 0.01) and, less likely to have access to basic necessities compared to other groups (*F* = 12.5, *p* = <0.0001). 57.5% (270/920) of adolescent mothers had experienced education interruption and were at least one school grade behind where they should be according to their age. Compared to mothers of children who were classified as HU, mothers of children who were classified as HEU were more likely to be at least one school grade behind (75.5% vs. 55.3%, *X*^2^ = 7.46, *p* = 0.02). Cash grant receipt was found to be high among all groups, although a trend was identified for households of HEU children to be more likely in receipt of grants (X^2^ = 5.83, *p* = 0.05). Household employment, perceived social support, and violence exposure among adolescent mothers were not found to differ according to child HIV status. For adolescent mothers living with HIV, 10.5% of mothers were perinatally infected with HIV and, mode of HIV infection (perinatal vs. postnatal) was similar among mothers of children living with HIV and HEU children (X^2^ = 0.02, *p* = 0.88). ART adherence (in the past week) was found to be significantly lower among mothers of children living with HIV compared to HEU children (36.4% vs. 78.7%, *p* = 0.001). One hundred and thirteen adolescent mothers (12.3%) scored above the cut-off on at least one measure of mental health symptomology. A trend was identified for mothers of HEU children to be more likely to report common mental disorder (17.5%, X^2^ = 5.95, *p* = 0.05, see Table 1).

**Table 1:** Maternal and child sociodemographic characteristics according to according to child HIV status (n = 920).

	Total sample (n = 920)	HIV (n = 11)	HEU (n = 189)	HU (n = 720)	*X2/F*, p-value
**Maternal characteristics**					
Age (years; mean [SD])	18.0 (1.6)	19.2 (1.7)^a^	19.2 (1.7)^a^	17.7 (1.5)	**76.2, <0.0001**
Age at birth of first child (years; mean [SD])	16.6 (1.5)	16.6 (1.5)	17.4 (1.4)^a^	16.3 (1.4)	**45.8, <0.0001**
Food secure (had food for the past week and can afford 3 meals)	656 (71.3%)	4 (36.3%)	128 (67.7%)	524 (72.8%)	**8.51, 0.01**
Number of necessities mother can afford (0–8; mean [SD])	5.2 (2.2)	4.0 (2.1)	4.6 (2.4)^a^	5.4 (2.2)	**12.5, <0.0001**
Access to basic necessities	181 (19.7%)	1 (9.1%)	31 (16.4%)	149 (20.7%)	2.53, 0.28
Home receives any grant	847 (92.1%)	10 (90.9%)	182 (96.3%)	655 (91.0%)	5.83, 0.05
Someone at home is employed and a grant is received	526 (57.2%)	6 (54.6%)	111 (58.7%)	409 (56.8%)	0.26, 0.88
Maternal education interruption (school grade behind)	270 (57.5%)	2 (66.7%)	37 (75.5%)	231 (55.3%)	**7.46, 0.02**
Social support (highest score for all domains: MOSS)	812 (88.3%)	10 (90.9%)	158 (83.6%)	644 (89.4%)	5.01, 0.08
Exposure to community violence	123 (13.4%)	2 (18.2%)	25 (13.2%)	96 (13.3%)	0.22, 0.89
Any domestic violence or domestic arguments	70 (7.6%)	2 (18.2%)	16 (8.5%)	52 (7.2%)	2.09, 0.35
*Mode of HIV infection (n = 200)*					0.02, 0.88
Perinatal HIV infection	21 (10.5%)	1 (9.1%)	21 (10.6%)	n/a	
Postnatal (recent) HIV infection	179 (89.5%)	10 (90.9%)	169 (89.4%)	n/a	
Adherence to ART in the last week (n = 200)	152 (76.4%)	4 (36.4%)	148 (78.7%)	n/a	**10.33, 0.001**
Any common mental disorder*	113 (12.3%)	1 (9.1%)	33 (17.5%)	79 (11.0%)	5.95, 0.05
**Child characteristics**					
Age (Months; Mean [SD])	17.9 (14.2)	28.5 (13.2)^a^	21.6 (16.0)^a^	16.8 (13.5)	**12.2, <0.0001**
Biological sex (female)	442 (48.0%)	6 (54.6%)	89 (47.1%)	347 (48.2%)	0.26, 0.88

*Any common mental disorder- defined as scoring above the cut-off for at least one mental health measure (depressive, anxiety, posttraumatic stress, suicidality symptomology)|Tukey ‘s HSD post hoc tests undertaken to identify mean differences between groups (for continuous variables only):

^a^ Statistically different from the HIV unexposed (HU) group (p = <0.05),

^b^ Statistically different from the HIV exposed uninfected (HEU) group (p = <0.05).

### Cognitive development of children born to adolescent mothers

**Fig 1:**
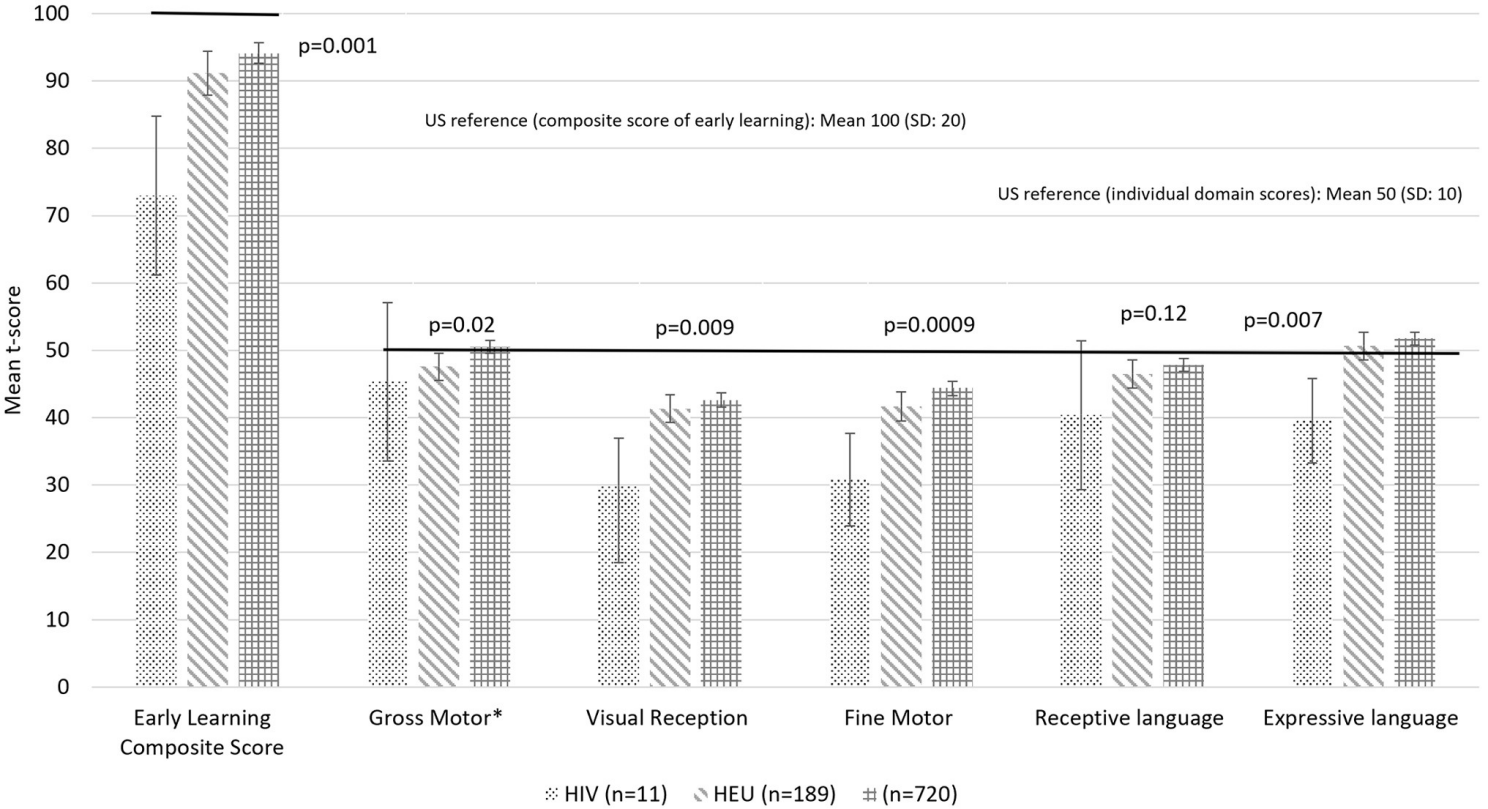
Cognitive development scores of children born to adolescent mothers according to child HIV status (n = 920). *Gross motor n = 831 (HIV = 9, HEU = 163, HU = 659) | Individual domain scores range from 20–80 | Early Learning Composite Score ranges from 49–155.

Table 2 and Fig 1 presents the cognitive develop scores of children according to child HIV status. The mean early years composite score for the sample was 93.2 (SD: 21.3), a score lower than expected compared to USA reference population utilised within the development of the scale (93.2 vs. 100, *t* = 9.67, *p* = <0.0001). Within crude analyses, average scores on all developmental domains (inclusive of the composite score, but exclusive of the language sub-domain) identified a stepwise pattern in which children classified as HU performed better than the children classified as HEU and, the children classified as HEU performed better than the children classified as living with HIV. Further analyses with post hoc testing identified that children who were HIV and HEU performed similarly on the gross motor domain and, HEU children scored significantly worse than HU children. Within the visual reception and fine motor domains and, within the overall composite score, children living with HIV performed worse than both HEU and HU children. Within the expressive language domain children living with HIV performed worse than HU children (see Table 2 and Fig 1).

**Table 2:** Cognitive development of children born to adolescent mothers according to child HIV status (n = 920).

Mullen Scales of Child Development (n = 920; Mean [SD])	Total sample (n = 920)	HIV (n = 11)	HEU (n = 189)	HU (n = 720)	*F*, p-value
Early Learning Composite Score	93.2 (21.3)	73.0 (17.6)^a^^,^^b^	91.2 (22.7)	94.1 (20.8)	**6.45, 0.001**
Gross Motor*	49.9 (12.5)	45.4 (15.3)	47.6 (13.5)^a^	50.5 (12.2)	**3.91, 0.02**
Visual Reception	42.1 (14.3)	29.9 (10.6)^a^^,^^b^	41.3 (14.2)	42.6 (14.3)	**4.77, 0.009**
Fine Motor	43.7 (14.5)	30.8 (10.2)^a^^,^^b^	41.7 (15.1)	44.4 (14.3)	**7.09, 0.0009**
Receptive language	47.4 (13.5)	40.4 (16.4)	46.5 (14.8)	47.8 (13.0)	2.14, 0.12
Expressive language	51.5 (13.3)	39.6 (9.3)^a^	50.7 (14.2)	51.8 (13.0)	**4.94, 0.007**

*Gross motor n = 831 (HIV = 9, HEU = 163, HU = 659)| Individual domain scores range from 20–80 | Early Learning Composite Score ranges from 49–155 | Tukey’s HSD post hoc tests undertaken to identify mean differences between groups:

^a^Statistically different from the HU group (p = <0.05),

^b^Statistically different from the HEU group (p = <0.05)

### Associations between child HIV status (living with HIV, HEU, HU) and child cognitive development

Table 3 presents linear regression models exploring the cross-sectional associations between child HIV status and composite scores of early learning. Within univariate analyses (model 1), a stepwise pattern was identified between child HIV status and the composite score for early learning i.e., children living with HIV were found to perform worse than HU children (*p* = <0.0001) and a trend was identified for HEU children to perform worse than HU children (*p* = 0.10). A similar pattern was identified within multivariate analyses adjusting for demographic characteristics (model 2; children living with HIV [*B* = -14.9, 95% CI: -26.9, -2.95, p = 0.02]). Model 3 presents potential characteristics mitigating the relationship between child HIV status and reduced child cognitive development scores. Within this multivariate model (model 3), maternal education interruption (being at least a school grade behind) was identified as a potential risk factor for reduced child cognitive development scores (*B* = -11.0 [95% CI:-14.9, -7.04], p = <0.0001) and, older maternal age (at birth of child) was found to be a protective factor, associated with increased child cognitive development scores (*B* = 1.79 [95% CI: 0.41, 3.17], p = 0.01). Analyses exploring individual cognitive domain scales can be found in S1 Table.

**Table 3:** Linear regression models exploring the relationship between child HIV status and child cognitive development among children born to adolescent mothers.

	Early Learning Composite Score	
	*B* (95% CI)	*p*
**Model 1. (Univariate model)** *		
HU (n = 720)	1 (Ref.)	
HEU (n = 189)	-2.84 (-6.24, 0.54)	0.10
HIV (n = 11)	-21.1 (-33.7, -8.46)	**<0.001**
Child age (months)	-0.51 (-0.60,-0.42)	**<0.0001**
Child biological sex (female)	-1.30 (-4.06, 1.45)	0.35
Number of necessities mother can afford (0–8)	0.47 (-0.14, 1.09)	0.13
Maternal education interruption (school grade behind)	-8.87 (-12.59, -5.15)	**<0.0001**
Maternal age at birth of child (years)	0.75 (-0.18, 1.68)	0.12
**Model 2. (Multivariate model adjusted for demographic characteristics)** *		
HU (n = 720)	1 (Ref.)	
HEU (n = 189)	-0.28 (-3.55, 2.99)	0.87
HIV (n = 11)	-14.9 (-26.9, -2.95)	**0.02**
**Model 3. (Multivariate model adjusted for potential mitigating factors)** *		
HU (n = 720)	1 (Ref.)	
HEU (n = 189)	4.78 (-1.31, 10.9)	0.12
HIV (n = 11)	-15.0 (-37.9, 7.85)	0.19
Maternal education interruption (school grade behind)	-11.0 (-14.9, -7.04)	**<0.0001**
Maternal age at birth of child (years)	1.79 (0.41, 3.17)	**0.01**

*Model 1. Univariate linear regression models | Model 2. Multivariate linear regression model. Covariates included within model 2: child age (months), child biological sex (female), number of necessities mother can afford (0–8) | Model 3. Exploratory multivariate linear regression model exploring possible mitigating factors for poor child cognitive development scores. Variables included in model 3: child HIV status, maternal education interruption, maternal age at birth of child (years)

## Discussion

This is the first known study exploring the association between child HIV status and child cognitive development outcomes for children born to adolescent mothers in South Africa. The HIV transmission rate from mother to child for the adolescents living with HIV was 5.5% (11/200). Crude analyses identify a stepwise pattern in relation to average child cognitive development scores according to child HIV status, with children living with HIV performing worse than children who were classified as HEU, and children who were HEU performing slightly worse than children who were classified as HU on the majority of child development domains. In multivariate analyses, children living with HIV disproportionately performed worse across domains of development explored compared to the HU children. Likewise, average scores for HEU children were lower than the HU group although this only reached significance in relation to gross motor skills. Exploratory multivariate analyses identified maternal education interruption as a potential risk factor to poorer child cognitive development and older maternal age (at the birth of their child) as a potential protective factor within this sample. Findings from this study support previous research highlighting the role of HIV and maternal factors on the developmental trajectories of children born to adult mothers and address a critical evidence gap regarding the cognitive profile of children born to adolescent mothers affected by HIV.

The crude HIV transmission rate from mother to child within this sample is seemingly above the South African national average (4.6%; n.b. confidence intervals are not available for this estimate) [43] and beyond global targets to eliminate mother to child transmission of HIV [44], highlighting adolescent mothers as a fundamental group within the HIV response. The promotion of vertical transmission prevention among adolescent mothers and adherence to ART would seemingly be beneficial to both adolescent mothers and their children, decreasing the number of children living with HIV within future generations and thus having potential benefits for child development outcomes. Overall, children born to adolescent mothers within the sample were found to perform worse on cognitive development tasks compared to the USA reference group from which the Mullen Scales of Early Learning were developed [40] and, children born to adult mothers within the sub-Saharan Africa region [45]. As such, these findings highlight the potential risks of adolescent pregnancy for the cognitive development of children born to adolescent mothers. These analyses support previous studies, highlighting differences in cognitive performance among children affected by HIV–living with HIV, HEU and HU–born to adult mothers [20]. Children living with HIV seemingly performed worse on cognitive development tasks compared to children classified as HEU and HU. As such, those children living with HIV are potentially most at risk for cognitive delay and, programmatic responses known to bolster development outcomes within similar settings may be of benefit (e.g., book sharing) [26]. However, tailoring to the specific needs of children born to adolescent mothers may be required. While it is encouraging that the difference in child development scores of children among children classified as HEU and HU was smaller than the difference between children living with HIV and those classified as HU, given the efforts to eliminate mother to child transmission of HIV, there are a growing number of children who are classified as HEU. As such, the promotion of cognitive development among this group should be remain an ongoing consideration to promote potential within future generations and it should be studied whether an early lack of developmental differences as found in the current study is retained as children grow older.

Exploratory analyses focusing on potential mitigating factors within the relationship between child HIV status and child cognitive development scores, highlighted maternal education interruption as being a potential risk factor for child cognitive development and older maternal age (at birth) as a potential protective factor for child cognitive development among children born to adolescent affected by HIV. Given the cross-sectional nature of these analyses, we are unable to ascertain whether education interruption preceded the pregnancy, and thus may have been a contributing factor to pregnancy, or if the interruption to education was a result of the pregnancy during adolescence. Nevertheless, the promotion of education continuity and school reenrolment following birth may be of benefit to both adolescent mother and their child. Whilst the South African Department of Education supports the continued education of pregnant girls and young mothers, at least a quarter of adolescents might discontinue school during the pregnancy [46] and a large proportion of young mothers does not manage to return to school postpartum [47–51]. Yet, there remains a clear lack of evidence-based programmes that successfully address adolescent mothers’ hurdles to return to school, for example through financial and childcare provisions [1, 52].

Within global literature, younger maternal age has previously been found to be associated worse development outcomes for children as well as harsher parenting practices and, less knowledge of child development [8–11], which may have implications for child development. These data suggest that children born to younger adolescent mothers may be at risk for worse cognitive outcomes and may require additional support. Such findings highlight the complexity of child cognitive development within this setting and the potential role of a broad range of factors within child development outcomes as both age of pregnancy and maternal education have been found to be associated with numerous factors inclusive of poverty, stigma, and violence exposure [53–55].

These data indicate that interventions should focus on several core factors. Firstly, continued efforts regarding the prevention of vertical transmission of HIV including ensuring good treatment adherence for adolescent mothers during pregnancy and the postpartum period. Such efforts would support global targets to eliminate mother to child transmission of HIV. Secondly, bolstering child cognitive development with existing interventions known to enhance development (e.g., early childhood stimulation and parenting interventions) may aid long term outcomes for children born to adolescent mothers affected by HIV. Thirdly, supporting adolescent mothers showing poor school performance might contribute to the positive cognitive development of their children. In addition to an enabling policy-environment, additional efforts that facilitate adolescents continued school enrolment during pregnancy and their timely return to school after birth are needed to bolster adolescent mother educational attainment. However, the bidirectional relationship between school dropout and adolescent pregnancy may require further research to understand the complexity of this relationship and its impact on child cognitive developments. Nevertheless, supporting school return following pregnancy for adolescent mothers would aid in bolstering adolescent mother educational attainment. Finally, sexual, and reproductive health interventions focusing on contraception and risk management may aid in assisting adolescents to delay pregnancy until they are older. Despite this, to ensure the prosperous development of future generations, the need of pregnant and parenting adolescents should be linked to adolescent pregnancy prevention efforts.

### Limitations

Findings should be interpreted within the context of some study limitations. Firstly, the data presented are cross-sectional and therefore the direction of causality for any identified associations cannot be established. Secondly, data presented are mostly self-report, including the assignment of groups relating to child HIV status. However, where possible, child medical records were utilised to confirm child HIV status. Thirdly, data is drawn solely from South Africa which may impact the generalisability of any findings. Fourthly, the child development scales utilising within analyses (Mullen Scales of Early Learning) were developed utilising a reference population within the USA which may limit the cultural validity of the assessments. However, these measures have previously been used extensively within sub-Saharan Africa [56] and have deemed to be effective within independent assessment of child cognitive development as well as preferable over caregiver reporting. Finally, the multivariate analyses undertaken as part of this study were exploratory and likely only present a segment of the complex mosaic of factors influencing the relationship between child HIV status and child cognitive development among children born to adolescent mothers in South Africa. For instance, while main caregiver characteristics were presented to place this research in context. The role of other caregivers within the development of children was not explored within the current study. Future research is required to wholly understand the complexity of the lives of adolescent mothers and their children and the impact different exposures on child cognitive development. Likewise, it was beyond the scope of this study to explore child cognitive development according to mode of maternal HIV infection. For context, mode of maternal HIV infection was ascertained from a derived variable and included within descriptive analyses. However, further research is required to wholly understand the multigenerational effects of HIV on child cognitive development. Furthermore, while it is encouraging to identify only a small sample of children living with HIV within the sample, the small sample size within this group limited the statistical analyses that could be undertaken as part of this study and may have diluted the effect of HIV status on cognitive development. Nevertheless, these analyses provide an initial step in addressing a critical void within the literature relating to the development of children born to adolescent mothers within the context of HIV within sub-Saharan Africa. Thus, providing a platform for future investigations into the experience of children born to adolescent mothers affected by HIV. Future studies would benefit from longitudinal analyses of groups with a more equal distribution of HIV status to gain further clarity regarding the factors influencing the cognitive development of children born to adolescent mothers affected by HIV as well as further analyses of the impact of HIV on development beyond the early developmental period and, comparisons with adult mother-child dyads in the region.

## Conclusions

These exploratory findings address a substantive evidence gap regarding the cognitive development of children born to adolescent mothers affected by HIV in South Africa and provide a foundation for future research regarding the children of adolescent mothers affected by HIV in sub-Saharan Africa. Child cognitive development scores were found to differ according to child HIV status. Compared to children who were classified as HU, children who were classified as HEU were found to perform worse on cognitive tasks and, children living with HIV were found to perform worse overall. Existing interventions to bolster child cognitive development may be beneficial however, adaptation for the specific needs of adolescent mothers and their children may be required. Attention to maternal continuity of education and delayed conception may be additional interventions to consider. Targeted interventions particularly among younger adolescent mothers and those with lower educational attainment may identify those families who may be most in need.

## Supporting information

S1 Table Linear regression models exploring the relationship between child HIV status and child cognitive development (individual development scales) among children born to adolescent mothers. (DOCX)
