# Supplementary material for: Adolescent parenthood and HIV-infection in South Africa—Associations with child cognitive development
Source: PLOS Glob Public Health. 2022 May 2;2(5):e0000238. doi: 10.1371/journal.pgph.0000238 (PMC10021425; doi:10.1371/journal.pgph.0000238)
Supplement: S1 Table — (DOCX) [file pgph.0000238.s001.docx]

**Supplementary Material**

S1 Table presents linear regression models exploring the cross-sectional associations between child HIV status and scores for individual developmental domains. Univariate analyses (model 1) present a similar stepwise pattern identified between child HIV status and individual developmental domain scores i.e., children living with HIV were found to perform worse across all developmental domains, reaching significance for visual reception, fine motor, and expressive language domains. This pattern was retained within multivariate analyses (model 2). With exploratory analyses exploring potential characteristics mitigating the relationship between child HIV status and individual developmental domain scores (model 3), maternal education interruption (being at least a school grade behind) was identified as a potential risk factor for reduced scores across all individual developmental domains. Older maternal age (at birth of child) was found to be associated with increased expressive language and receptive language scores (see S1 Table).

**S1 Table. Linear regression models exploring the relationship between child HIV status and child cognitive development (individual development scales) among children born to adolescent mothers**

|  | **Gross motor skills** | | **Visual reception** | | **Fine motor** | | **Receptive language** | | **Expressive language** | |
| --- | --- | --- | --- | --- | --- | --- | --- | --- | --- | --- |
|  | ***B* (95% CI)** | ***p*** | ***B* (95% CI)** | ***p*** | ***B* (95% CI)** | ***p*** | ***B* (95% CI)** | ***p*** | ***B* (95% CI)** | ***p*** |
| **Model 1. (Univariate model) *** | | | | | | | | | | |
| HU (n=720) | 1 (Ref.) |  | 1 (Ref.) |  | 1 (Ref.) |  | 1 (Ref.) |  | 1 (Ref.) |  |
| HEU (n=189) | -2.83  (-4.97, -0.68) | 0.01 | -1.32  (-3.60, 0.96) | 0.26 | -2.70  (-5.01, -0.39) | **0.02** | -1.26  (-3.42, 0.90) | 0.25 | -1.08  (-3.20, 1.04) | 0.32 |
| HIV (n=11) | -5.02  (-13.24, 3.21) | 0.23 | -12.6  (-21.1, -4.18) | **0.003** | -13.6  -22.2, -5.03) | **0.002** | -7.32 (-15.3, 0.71) | **0.07** | -12.2  (-20.1, -4.29) | **0.003** |
| Child age (months) | -0.23  (-0.31, -0.15) | **<0.0001** | -0.23  (-0.30, -0.17) | **<0.0001** | -0.23  (-0.30, -0.17) | **<0.0001** | -0.32  (-0.38, -0.27) | **<0.0001** | **-0.28**  **(-0.34, -0.22)** | **<0.0001** |
| Biological sex (female) | -0.81  (-2.52, 0.89) | 0.35 | 0.27  (-1.5, 2.12) | 0.77 | -0.83  (-2.70, 1.05) | 0.39 | -1.39  (-3.14, 0.35) | 0.12 | -0.08  (-2.54, 0.90) | 0.35 |
| Number of necessities mother can afford (0-8) | 0.03  (-0.35, -0.42) | 0.87 | 0.30  (-0.11, -0.71) | 0.16 | 0.26  (-0.16, 0.68) | 0.22 | 0.29  (-0.10, 0.68) | 0.15 | 0.15  (-0.23, 0.54) | 0.44 |
| Maternal age at birth of child (years) | -0.05  (-0.63, 0.54) | 0.88 | 0.26  (-0.37, 0.89) | 0.41 | 0.50  (-0.14, 1.14) | 0.12 | 0.32  (-0.27, 0.92) | 0.29 | 0.54  (-0.04, 1.13) | 0.07 |
| Maternal education interruption (school grade behind) | -2.52  (-4.79, -0.25) | **0.03** | -6.12  (-8.61, -3.6) | **<0.0001** | -2.84  (-5.38, -0.29 | **0.03** | -4.29  (-6.63, -1.96) | **<0.0001** | -5.04  (-7.34, -2.73) | **<0.0001** |
| **Model 2. (Multivariate model adjusted for demographic characteristics) *** | | | | | | | | | | |
| HU (n=720) | 1 (Ref.) |  | 1 (Ref.) |  | 1 (Ref.) |  | 1 (Ref.) |  | 1 (Ref.) |  |
| HEU (n=189) | -2.23  (-4.38, -0.07) | **0.04** | -0.07  (-2.34, 2.20) | 0.95 | -1.57  (-3.87, 0.74) | 0.18 | 0.41  (-1.66, 2.49) | 0.70 | 0.28  (-1.80, 2.35) | 0.79 |
| HIV (n=11) | -3.01  (-11.2, 5.15) | 0.47 | -9.72  (-18.0, -1.41) | **0.02** | -10.9  (-19.4, -2.46) | **0.01** | -3.34  (-11.0, 4.27) | 0.39 | -8.90  (-16.5, -1.30) | **0.02** |
| **Model 3. (Multivariate model adjusted for potential mitigating factors) *** | | | | | | | | | | |
| HU (n=720) | 1 (Ref.) |  | 1 (Ref.) |  | 1 (Ref.) |  | 1 (Ref.) |  | 1 (Ref.) |  |
| HEU (n=189) | 1.96  (-1.85, 5.76) | 0.31 | 3.10  (-0.99, 7.20) | 0.14 | 2..53  (-1.66, 6.72) | 0.24 | 2.70  (-1.13, 6.52) | 0.17 | 1.32  (-2.45, 5.10) | 0.49 |
| HIV (n=11) | -1.18  (-14.9, 12.6) | 0.87 | -4.41  (-19.8, 11.0) | 0.57 | -13.9  (-29.7, 1.82) | 0.08 | -3.89  (-18.3, 10.5) | 0.60 | -8.63  (-22.83, 5.57) | 0.23 |
| Maternal education interruption (school grade behind) | -2.60  (-5.05, -0.14) | **0.04** | -7.33  (-9.97, -4.68) | **<0.0001** | -3.54  (-6.25, -0.84) | **0.01** | -5.59  (-8.06, -3.12) | **<0.0001** | -6.28  (-8.72, -3.84) | **<0.0001** |
| Maternal age at birth of child (years) | -0.06  (-0.95, 0.83) | 0.90 | 0.99  (0.05, 1.92) | **0.04** | 0.55  (-0.40, 1.50) | 0.26 | 1.11  (0.24, 1.98) | **0.01** | 1.18  (0.32, 2.04) | **0.007** |

NB. Missing data for Gross motor skills scores: HIV=9, HEU=163, HU=659 | *Model 1. Univariate linear regression models | Model 2. Multivariate linear regression model. Covariates included within model 2: child age (months), child biological sex (female), number of necessities mother can afford (0-8) | Model 3. Exploratory multivariate linear regression model exploring possible mitigating factors for poor child cognitive development scores. Variables included in the model: child HIV status, maternal education interruption, maternal age at birth of child (years)
